# Liposome Extract of Stachys pilifera Benth Effectively Improved Liver Damage due to Bile Duct Ligation Rats

**DOI:** 10.1155/2021/8141563

**Published:** 2021-10-18

**Authors:** Zahra Moslemi, Hassan Bardania, Izadpanah Gheitasi, Zahra Barmoudeh, Navid Omidifar, Hamidreza Parvin, Bahman Khalvati, Mohamad Hassan Fouani, Mohsen Alipour, Amir Hossein Doustimotlagh

**Affiliations:** ^1^Student Research Committee, Yasuj University of Medical Sciences, Yasuj, Iran; ^2^Medicinal Plants Research Center, Yasuj University of Medical Sciences, Yasuj, Iran; ^3^Cellular and Molecular Research Center, Yasuj University of Medical Sciences, Yasuj, Iran; ^4^Clinical Research Development Unit, Imamsajad Hospital, Yasuj University of Medical Sciences, Yasuj, Iran; ^5^Biotechnology Research Center, Department of Pathology, School of Medicine, Shiraz University of Medical Sciences, Shiraz, Iran; ^6^Pharmaceutical Science Research Center, School of Pharmacy, Shiraz University of Medical Sciences, Shiraz, Iran; ^7^Department of Nanobiotechnology, Faculty of Biological Sciences, Tarbiat Modares University, Tehran, Iran; ^8^Department of Advanced Medical Sciences & Technologies, School of Medicine, Jahrom University of Medical Sciences, Jahrom, Iran

## Abstract

Herbal medicines harbor essential therapeutic agents for the treatment of cholestasis. In this study, we have assessed the anticholestatic potential of *Stachys pilifera Benth*'s (SPB's) hydroalcoholic extract encapsulated into liposomes using bile duct ligation- (BDL-) induced hepatic cholestasis in rats. Aspartate aminotransferase (AST), alanine aminotransferase (ALT), alkaline phosphatase (ALP), malondialdehyde (MDA), total thiol (T-SH) content, protein carbonyl (PCO), total bilirubin (TBIL), albumin (ALB), and nitric oxide (NO) metabolite levels were measured in either liver tissue or plasma to assess liver damage. Moreover, expression of proinflammatory cytokines (IL-1*β* and TNF-*α*) and liver fibrosis markers (TGF-*β* and SM-*α*) which are driving forces of many liver disorders was also determined. The activity of AST, ALT, and ALP was significantly enhanced in the BDL group in comparison to the control group; however, treatment with liposomal (SPB) hydroalcoholic extract significantly reduced AST and ALT's activity. Increases in MDA, TBIL, and NO levels and T-SH content due to BDL were restored to control levels by liposomal (SPB) hydroalcoholic extract treatment. Similarly, hepatic and plasma oxidative marker MDA levels, significantly enhanced by BDL, were significantly decreased by liposomal (SPB) hydroalcoholic extract treatment. Moreover, histopathological findings further demonstrated a significant decrease in hepatic damage in the liposomal (SPB) hydroalcoholic extract-treated BDL group. In addition, liposomal (SPB) hydroalcoholic extract treatment decreased the liver expression of inflammatory cytokines (IL-1*β*, TNF-*α*) and liver fibrosis markers (TGF-*β* and SM-*α*). Since liposomal (SPB) hydroalcoholic extract treatment alleviated the BDL-induced injury of the liver and improved the hepatic structure and function more efficiently in comparison to free SPB hydroalcoholic extract, probable liposomal (SPB) hydroalcoholic extract exhibits required potential therapeutic value in protecting the liver against BDL-caused oxidative injury.

## 1. Introduction

Nowadays, millions of people suffer from chronic or acute liver disorders [1]. Hepatic cholestasis is a common pathophysiological disorder that leads to the toxic build-up of biliary salts in the liver [2]. In case not treated, chronic cholestasis will lead to hepatic cirrhosis, fibrosis, and eventually liver failure necessitating liver transplantation [3, 4]. The mechanism of fibrosis in liver cholestasis is not well understood [5]; various factors such as oxidative stress, transport disorders, immune suppression, and inflammatory injury can cause cholestasis [6]. Previous studies have demonstrated that oxidative stress or free radicals lead to liver injury and cholestasis [7]. The accumulation of biliary salts in the hepatic membrane increases the reactive oxygen species (ROS) content and subsequently causes oxidative stress leading to cell destruction [8, 9]. In severe liver injury, neutrophils and Kupffer and hepatic cells produce ROS. Moreover, increased ROS production damages cellular macromolecules such as proteins, lipids, and DNA [10]. Pathophysiological conditions induced by bile duct ligation (BDL) resemble those exhibited by cholestasis, where it causes elevation of bile acid concentration and lipid peroxidation, as well as stimulation of inflammatory cells and phagocytic capacity of polymorphonuclear leukocytes [11]. Over the past decades, anticholestatic potentials of medicinal herbs have gained extensive attention [6, 9].

Stachys pilifera Benth (SPB) belongs to the genus Stachys and the family of Lamiaceae. It grows in subtropical and tropical regions. Several phytochemical compounds have been isolated from Stachys species, namely, flavonoids, diterpenes, saponins, phenylethanoid glycosides, steroids, and terpenoids [12]. Moreover, biological studies have demonstrated that extracts of some Stachys species exhibit antibacterial, anti-inflammatory, antitoxic, antihepatitis, and antioxidant effects [13]. *SPB* is used in Iranian traditional medicine to treat diseases such as infections, asthma, and rheumatoid arthritis [14]. Previous studies have confirmed the antioxidant properties of SPB in hepatotoxicity and nephrotoxicity induced by carbon tetrachloride and cisplatin, respectively [14, 15]. Today, different nanocarriers such as liposomes are used to encapsulate and deliver various constituents of plants. Liposomes with biocompatibility and biodegradability properties could encapsulate both hydrophobic and hydrophilic compounds [16, 17]. Therefore, they have high efficacy to envelop plant extracts containing many hydrophobic and hydrophilic compounds. The current investigation was performed to assess the efficacy of *SPB*'s hydroalcoholic extract encapsulated into liposomes on hepatic cholestasis induced by BDL in male rats.

## 2. Materials and Methods

### 2.1. Preparation of *SPB*'s Hydroalcoholic Extract

In the current investigation, aerial parts of *SPB* were collected from Yasuj, Iran. Gathered plant specimens were authenticated by Dr. Azizollah Jafari, a botanist of Yasuj University. Plant samples were cleaned after collection and dried under appropriate conditions, away from sunlight. Thus, 0.1 kg of powdered plant was mixed with 1 L of solvent (70% ethanol). Subsequently, the mixture was stored at 37°C for two days and later on filtered with Whatman filter paper No. 1. Then, the extract was concentrated and gathered using a vacuum evaporator. Finally, the extract was dehydrated and stored at -20°C.

### 2.2. Chemicals

Trichloroacetic acid (TCA), 5,5′-dithiols-(2-nitrobenzoic acid) (DTNB), 2,4,6-tris(2-pyridyl)-s-triazine (TPTZ), dipalmitoylphosphatidylcholine (DPPC), cholesterol, and thiobarbituric acid (TBA) were purchased from Sigma (St. Louis, MO, USA). 2,4-Dinitrophenylhydrazine (DNPH) and formaldehyde were provided from Merck (Germany). All other reagents and chemicals used were of analytical grade.

### 2.3. Liposome Preparation

SPB extract encapsulation into liposomes was performed using the thin film hydration method [18–20]. A dry, thin lipid film was prepared by evaporating the chloroform/methanol (50 : 50 *v*/*v*) from dipalmitoylphosphatidylcholine (DPPC) : cholesterol : SPB extract (7 : 0.7 : 3 weight ratio, respectively) solution by a rotary evaporator (Heidolph, Germany). Subsequently, hydration of the film was performed by phosphate buffer saline (PBS) for 120 mins in a rotary evaporator. Next, the liposomes were sonicated for 3 min (20 s on and 10 s off for each cycle) with a probe sonicator (UP400S ultrasonic processor, Hielscher, Germany) at amplitude 60%; the implementation of ice-water bath inhibited temperature elevation. After that, size and morphology of liposomal formulation were studied using transmission electron microscopy (TEM) (JEM-1010; JEOL, Tokyo, Japan) and dynamic light scattering (DLS) (Malvern Zetasizer 3000) (Malvern Instruments, UK).

### 2.4. Animals

This study was conducted on 60 male Wistar rats (225 ± 25 gr). This study received approval from the Ethics Committee of Yasuj University of Medical Sciences (Code: IR.YUMS.REC.1399.021). Rats were housed under standard laboratory conditions (temperature (22 ± 2°C), humidity (55 ± 5%), and lighting (12/12 dark-light cycle)) and air system filtration (10-20 ventilations/hour) with free access to rat chow diet and water. Rats were treated according to the *Guidelines for the Care and Use of Laboratory Animals* (NIH Publication No. 86-23).

### 2.5. Experimental Procedure

Cholestasis was induced by bile duct ligation (BDL) in rats [21]. Individual rats were anesthetized by intraperitoneal injection of ketamine (50 mg/kg) and xylazine (10 mg/kg). Under sterile situations, in experimental groups, the common bile duct was tied from both sides and cut in half, causing an obstruction in bile secretion and cholestasis subsequently. Simultaneously, all surgeries were performed on sham control rats, except the closure of the bile duct. A total of 60 Wistar rats were divided into 7 groups, 6 rats in the sham control (SC) group and 9 rats in each BDL group. The SC, SC+Lip, and SC+SP groups were treated with normal saline, free liposome, and free SPB extract, respectively. The BDL, BDL+Lip, BDL+SP, and BDL+LSP groups were treated with normal saline, free liposome, free SPB extract, and SPB extract-loaded liposomes, respectively. One day after surgery, rats received SPB extract-loaded liposomes (0.5 g/kg body weight) or free SPB extract (500 mg/kg body weight) [12] by intraperitoneal injection over a 7-day period. In the end, rats were deeply anesthetized and subsequently killed by heart puncturing. Whole blood was collected in heparinized tubes. Liver tissue was detached and divided into two sections, one part was snap frozen in liquid nitrogen and stored at -70°C to be homogenized later on, and the other part was fixed in 10% formalin for liver histology. Plasma was separated from whole blood by centrifugation (3000 × g,10 minutes).

### 2.6. Biochemical Examination

Plasma was used to assess the activity of important liver enzymes, namely, alkaline phosphatase (ALP), alanine aminotransferase (ALT), aspartate aminotransferase (AST), total bilirubin (TBIL), and albumin (ALB). Measurements were recorded using diagnostic kits (Bionik Diagnostic Co., I.R., Iran) and in accordance with the manufacturer's specifications; samples were read using an automatic biochemical analyzer (Roche COBAS, Mira Plus, USA).

### 2.7. Measurement of Oxidative Stress Markers

Total antioxidant capacity (TAC) of plasma was assessed using the FRAP assay according to the Benzie and Strain method [22]. Malondialdehyde (MDA) levels, as an index of lipid peroxidation, were determined by spectrophotometric measurement of the MDA-thiobarbituric acid (MDA-TBA) complex formation after interacting with thiobarbituric acid (TBA) [23]. Protein carbonyl (PCO) concentrations were assayed colorimetrically through the interaction of carbonyl groups and DNPH [12]. Total thiol (tSH) content was measured utilizing a spectrophotometric technique according to the yellow color produced by the reaction of thiol groups and 5,5′-dithiobis (2-nitrobenzoic acid) DTNB [12]. Nitrite concentration in the samples was then measured by the Griess reaction [24].

### 2.8. Histopathological Examination

Liver sections fixed in 10% formalin were dehydrated in an ascending dilution series of ethanol (50, 70, and 100%), followed by paraffin wax embedding. Subsequently, tissue samples were cut into 3-4 *μ*m sections using a rotary microtome and stained with hematoxylin-eosin (HE) according to standard protocols; slides were surveyed by a pathologist in a blinded way.

### 2.9. Assessment of mRNA Using Real-Time PCR

Real-time PCR was implemented to assess the expression levels of inflammatory cytokines (*IL-1* and *TNF-α*), liver fibrosis markers (*TGF-β* and *SM-α*), and the housekeeping gene internal control (*β-actin*). Using the TRIzol reagent (Invitrogen, Carlsbad, CA), total RNA extraction was performed which was then reverse transcribed into cDNA using Sinaclon (Tehran, Iran). In order to quantify the expression of the target genes, synthesized cDNA was then subjected to real-time PCR on a Rotor Gene 3000 (Bio-Rad, USA); primers' sequences used in this study are listed in Table 1. The relative expression of each gene was calculated using the 2^–*Δ*Ct^ method.

### 2.10. Statistical Analysis

Data were analyzed using SPSS version 18 software. All data are described as the mean ± SEM. To calculate the statistical changes in quantitative variables between the studied groups, one-way ANOVA was done by Tukey's post hoc test. The significance level of *P* value ≤ 0.05 was determined for all tests.

## 3. Results

### 3.1. Characteristic of Liposomal Extract

**Figure 1:**
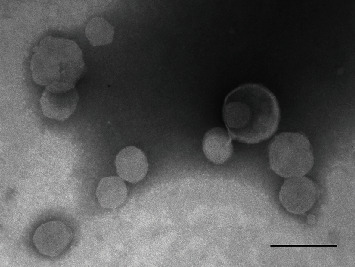
TEM image of liposomal SP extract; scale bar = 200 nm.

TEM results showed approximate size 117.84 ± 24 nm for liposomal extract with spherical form (Figure 1). In addition, measured size for liposomal extract by DLS analysis was about 173 nm which is significantly greater than TEM size, because of the fact that DLS analysis measures hydrodynamic size of nanoparticles.

### 3.2. Biochemical Parameters

**Figure 2:**
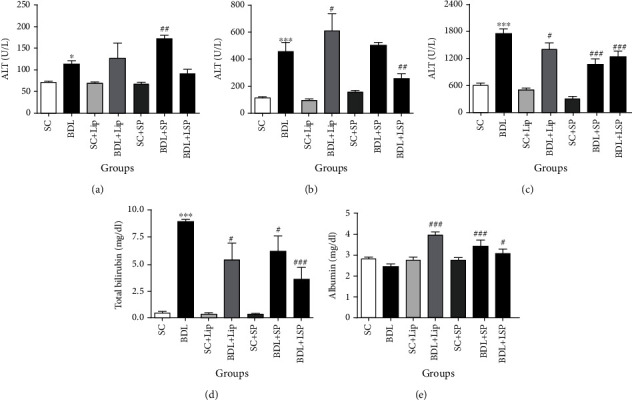
Effect of SP formulations on biochemical markers in BDL-induced cholestatic rats. Each value depicts the mean ± SEM. SC: sham group; BDL-alone: untreated bile duct-ligated rats; SP: *Stachys pilifera*; Lip: liposome; LSP: liposomal SP; ALT: alanine aminotransferase; AST: aspartate aminotransferase; ALP: alkaline phosphatase; TBIL: total bilirubin; ALB: albumin. ^∗^Significantly different from SC; ^∗^*P* value ≤ 0.05; ^∗∗∗^*P* value < 0.001. ^#^Significantly different from BDL-alone group, ^#^*P* value ≤ 0.05, ^##^*P* value < 0.01, and ^###^*P* value < 0.001.

Figure 2 depicts plasma levels of AST, ALT, ALP, and TBIL, which were significantly (*P* ≤ 0.05) higher in BDL rats in comparison to the SC group. Moreover, ALB content was significantly (*P* ≤ 0.05) reduced in BDL rats as compared to the SC group. BDL rats exposed to SPB's hydroalcoholic extract in its free or liposomal form caused a significant (*P* ≤ 0.05) decrease in ALP and TBIL levels, as well as a marked increase in ALB content in comparison to the BDL group. Furthermore, BDL rats treated with liposomal-SPB hydroalcoholic extract markedly reduced AST activity in comparison to the BDL group (*P* ≤ 0.05).

### 3.3. Liver Oxidative Stress Markers

**Figure 3:**
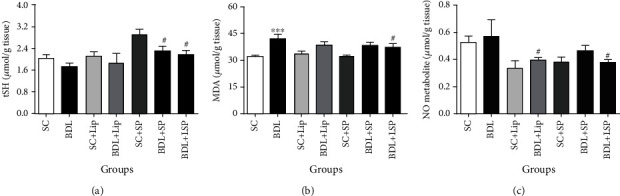
Effect of SP formulations on liver oxidative stress markers in BDL-induced cholestatic rats. Each value depicts the mean ± SEM. SC: sham group; BDL-alone: bile duct-ligated rats; SP: *Stachys pilifera*; Lip: liposome; LSP: liposomal SP; t-SH: total thiol; MDA: malondialdehyde; NO metabolites: nitric oxide metabolites. ^∗^Significantly different from SC, ^∗∗∗^*P* value < 0.001. ^#^Significantly different from BDL-alone group, ^#^*P* value ≤ 0.05.

Our results demonstrated that the BDL group, when compared to the SC group, exhibits slight reduction in tSH content and significant increase in MDA levels (Figure 3). Exposure of BDL rats to SPB hydroalcoholic extract significantly augmented t-SH content when compared to untreated BDL rats, while treatment with liposomal SPB hydroalcoholic extract not only increased t-SH content but also reduced MDA and NO metabolite in comparison to untreated BDL rats.

### 3.4. Plasma Oxidative Stress Markers

**Figure 4:**
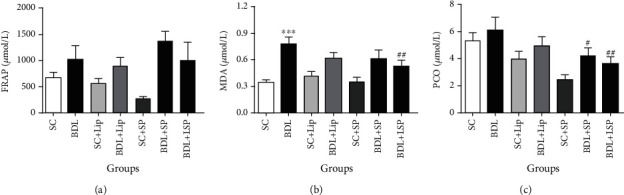
Effect of *SPB* formulations on plasma oxidative stress markers in BDL-induced cholestatic rats. Each value depicts the mean ± SEM. SC: sham group; BDL-alone: bile duct-ligated rats; SP: *Stachys pilifera*; Lip: liposome; LSP: liposomal SP; FRAP: ferric reducing antioxidant power; MDA: malondialdehyde; PCO: protein carbonyl. ^∗^Significantly different from SC, ^∗∗∗^*P* value < 0.001. ^#^Significantly different from the BDL-alone group, ^#^*P* value ≤ 0.05, ^##^*P* value < 0.01.

When compared to the SC group, the BDL group exhibited a slight increment in the plasma levels of FRAP and PCO, whereas MDA content was significantly increased (Figure 4). Administration of the liposomal hydroalcoholic extract of SPB at a dose of 0.5 g/kg significantly lowered MDA levels and PCO content in comparison to the BDL group (*P* ≤ 0.05). However, BDL rats treated with hydroalcoholic extract of SPB in the free form significantly ameliorated the PCO level in comparison to BDL rats (*P* ≤ 0.05).

### 3.5. Liver Histopathology

**Figure 5:**
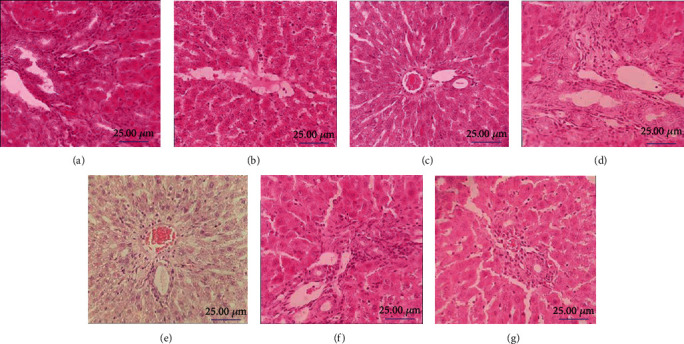
Light microscopy image of H&E-stained liver tissue slide (10x): (a) sham control (SC) rat; (b) bile duct-ligated (BDL) rat; (c) SC rat treated with liposome; (d) BDL rat treated with liposome; (e) SC rat treated with *Stachys pilifera* (*SP*): (f) BDL rat treated with SP; (g) BDL rat treated with liposomal extract. Scale bar: 25 *μ*m.

Hepatic cells with well-defined and normal cellular structures were clearly observed in liver samples of SC rats. The BDL group showed mild degeneration of hepatic cells, mild inflammation, and extensive bile duct proliferation in comparison to SC groups (Figure 5). Treatment with free *SPB* extract could reduce hepatocellular damage; however, administration of hydroalcoholic extract of SP in the liposomal form not only reduced hepatocellular damage but also markedly ameliorated portal inflammation and bile duct proliferation (Table 2).

### 3.6. Assessment of Inflammation and Liver Fibrosis Genetic Markers

**Figure 6:**
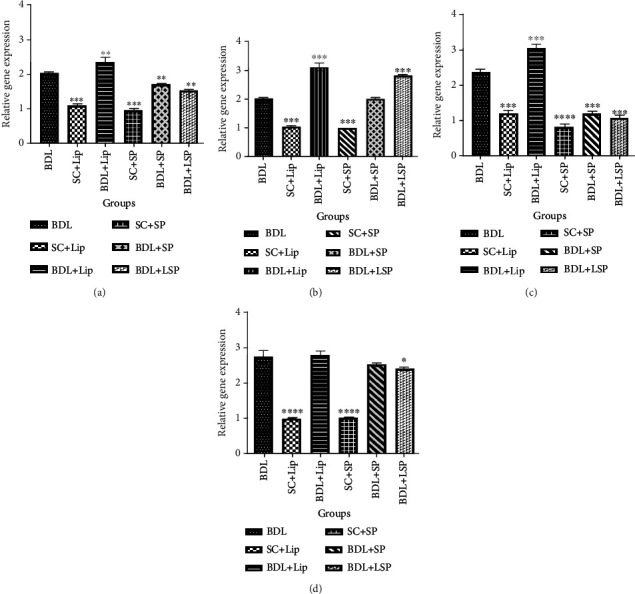
Expression rate of IL-1 (a), TNF-*α* (b), TGF-*β* (c), and SM-*α* (d). ^∗^*P* value < 0.05, ^∗∗^*P* value < 0.01, ^∗∗∗^*P* value < 0.001, and ^∗∗∗∗^*P* value < 0.0001 in comparison to the BDL group. BDL-alone: bile duct-ligated rats; Lip: liposome; SC: sham control; SP: *Stachys pilifera*.

In order to further investigate the efficacy of liposomal *SPB* extract, we assessed the expression levels of inflammatory cytokines (*IL-1* and *TNF-α*) and liver fibrosis genetic markers (*TGF-β* and *SM-α*). In comparison to the SCgroup, the relative expression of all examined genetic biomarkers was significantly elevated in all BDL groups. Moreover, an upregulation in TNF*-α* gene expression was observed upon the administration of *SPB* extract in the liposomal form (Figure 6). Furthermore, no significant variation in the expression levels of *α*-SMA gene was observed in all treated (BDL) groups when compared to the BDL group (Figure 6). However, the administration of *SPB* extract in the liposomal form significantly downregulated the expression rate of both *TGF-β* and *IL-1*, where this downregulation was more prominent in the expression of *TGF-β*.

## 4. Discussion

Cholestasis is a hepatic disorder that, if left untreated and allowed to progress, can lead to fibrosis, cirrhosis, and susbsequently death [25]. Bile acids accumulate in the liver following BDL; then, the buildup of toxins and mediators such as oxidative stress markers and inflammatory response leads to cell death and fibrosis [26]. Liposomes are spherical vesicles with one or more lipid layers. The origin of these lipid membranes may be natural or synthetic lipids. These nanoparticles can encapsulate and translocate hydrophilic compounds in their aqueous core or hydrophobic compounds in their lipid bilayers [16]. Moreover, biocompatibility and biodegradability of these nanoparticles have rendered these nanoparticles unique drug delivery vehicles. Liposomes can range in size from about 10 nanometers to a few micrometers, depending on the fabrication method [27–29]. The encapsulation of medicinal herbs into liposomes enhances their solubility, uptake, pharmacological efficacy, and circulation time in blood [30]. In traditional medicine, *SPB* is used to treat various disorders. Various studies have demonstrated the antitumor, antimicrobial, and antioxidant effects of *SPB* [13]. In this study, the antioxidant capacity of free and liposomal *SPB* was investigated in the BDL-induced cholestatic rats.

ALT and AST belong to the group of transaminases that catalyze the reversible transfer of amino groups between amino acids and alpha-acids. ALT is an enzyme found in liver cells and is implemented in the diagnosis of liver damage. AST is present in both the cytoplasm and the mitochondria of cells [31]. In cases of mild tissue damage, AST is mainly localized in the cytoplasm and a small amount is found in the mitochondria. However, severe tissue damage leads to the secretion of large amounts of AST from the mitochondria [32]. ALP is mainly attached to the cell membrane; it removes the phosphate groups from phosphate-containing organic esters as well as facilitates the transportation of molecules across the cell membrane [33]. In agreement with previous studies, our findings demonstrated that BDL caused liver damage through the assessment of AST, ALT, ALP, and total TBIL levels; see [34–36]. Moreover, histopathological findings further demonstrated that BDL caused liver damage. Furthermore, our findings showed that the liposomal form of the *SPB* extract is more effective than free *SPB* extract in ameliorating liver injury. Consistent with our results, Mansourian et al. showed that the methanolic extract of *SPB* significantly reduced ALT and AST in acetaminophen-induced toxicity [12].

It has been well recognized that an increase in lipid peroxides considerably disrupts the antioxidant defense mechanisms and is also associated with cell necrosis. Increased levels of MDA are an indicator of the amount of tissue lipid peroxidation, as well as a marker of tissue damage [2]. Several reports indicate that MDA levels increase after BDL in rats [2, 37]. Our results demonstrated that BDL rats exhibit elevated levels of hepatic and plasma MDA; however, liposomal *SPB* extract significantly reduced MDA levels. It seems that liposomal *SPB* extract exerts its protective effects via its antioxidant or free radical scavenging activity. Nitric oxide, a highly reactive mediator, is released by nonparenchymal liver cells (e.g., Kupffer cells and endothelial cells) in response to different stimuli in the liver [26]. Previous studies have shown that NO levels significantly increased in BDL rats [26, 38]. In comparison to the BDL group, administration of liposomal *SPB* extract resulted in a remarkable decrease in NO levels.

t-SH, another sensitive indicator of oxidative stress, plays an important role in the neutralization of free radicals. Consistent with our previous study [12], our results showed that treatment with free *SPB* hydroalcoholic extract or liposomal *SPB* extract markedly increased liver t-SH content, which might be attributed to SPB's intrinsic antioxidant capacity and inhibition of free radical formation.

Measurement of the PCO amount is one of the common methods for assessing protein damage by oxidation. Oxidative damage affects the structure and function of proteins [39]. Terzioglu et al. demonstrated that the PCO level significantly increases in the BDL group [39]. Results in our study showed that reduction of PCO content in the BDL group was more effective in liposomal *SPB* extract than free *SPB* extract. Inhibition of protein oxidation may be attributed to the high amount of flavonoids present in the SP extract.

Baradaran et al. showed that nanophytosomes can enhance antioxidant properties. Nanophytosomes protect curcumin and enhance its absorption. Curcumin nanophytosomes probably enhance the action of antioxidant enzymes and/or reduce oxidative stress [40]. In another study, Tan et al. examined the antioxidant activity of carotenoids encapsulated in liposome delivery vehicles, and their results showed that encapsulation could improve radical inhibitory activity of carotenoids [41].

Inflammation and Kupffer cells play a central role in the aggravation of liver fibrosis. Kupffer cells release proinflammatory cytokines such as TNF-*α*, IL-6, and IL-1, resulting in the activation of hepatic satellite cells, a hallmark event in liver fibrosis [42]. Moreover, cytokines trigger and activate hematopoietic stem cells to produce collagen. In accordance with our results, Sheen et al. [43] and Tahan et al. [44] have previously demonstrated that hepatic TNF-*α* expression, both at the mRNA and protein levels, was markedly elevated in BDL rats. In this study, the administration of SBP significantly downregulated the expression of TNF-*α* in BDL rats. In accordance with these results, Rabani et al. [45] demonstrated that SBP administration decreased TNF-*α* levels in paracetamol-induced nephrotoxicity. Based on anti-inflammatory activity of SBP, probable downregulation of TNF-*α* expression could be attributed to its anti-inflammatory effects.

## 5. Conclusion

Our findings showed that liposomal form of *SPB* extract is more effective than free *SPB* extract in ameliorating liver injury through reducing biochemical markers, improving histological changes, and favoring oxidant-antioxidant balance. Nanotechnology may offer innovative methods that have unlimited potentials for the prevention and treatment of cholestatic disease.

## Figures and Tables

**Table 1:** Sequences of primers used in the current study.

Gene	Primers	Primer sequence	Product size
GAPDH	Forward	5′-AGGTCGGTGTGAACGGATTTG-3′	123
	Reverse	5′-TGTAGACCATGTAGTTGAGGTCA-3′	
TNF-*α*	Forward	5′-GGTGATCGGTCCCAACAAGGA-3′	173
	Reverse	5′-CACGCTGGCTCAGCCACTC-3′	
IL-1*β*	Forward	5′-CACCTCTCAAGCAGAGCACAG-3′	79
	Reverse	5′-GGGTTCCATGGTGAAGTCAAC-3′	
TGF-*β*	Forward	5′-CCAGAGTGGCTGAACAACGG-3′	74
	Reverse	5′-GCGCTGGGTTGGAGATGTTAGG-3′	
*α*-SMA	Forward	5′-CCACATACATGGCAGGGACATTG-3′	174
	Reverse	5′-GGTACTGGGACGACATGGAAAAG-3′	

**Table 2:** Effect of SP formulations on liver histopathological markers in BDL-induced cholestatic rats.

Groups	Hepatocellular damage	Portal inflammation	Vasculature	Bile duct proliferation
SC	NL	NL	NL	NL
BDL	Mild degeneration	Mild inflammation	NL	Severely increased
SC+Lip	NL	NL	NL	NL
BDL+Lip	NL	Moderate inflammation	NL	Severely increased
SC+SPB	NL	NL	NL	NL
BDL+SPB	NL	Mild inflammation	NL	Moderately to severely increased
BDL+Lip+SPB	NL	NL	NL	Mildly increased

SC: sham control; BDL-alone: bile duct-ligated rats; SPB: *Stachys pilifera*; Lip: liposome; LSP: liposomal SP; NL: normal.

## Data Availability

The data used to support the findings of this study are included within the article.
